# Comparison between rapid and slow palatal expansion: evaluation of selected periodontal indices

**DOI:** 10.1186/1746-160X-10-30

**Published:** 2014-08-15

**Authors:** Stefano Mummolo, Enrico Marchetti, Francesca Albani, Vincenzo Campanella, Filippo Pugliese, Salvatore Di Martino, Simona Tecco, Giuseppe Marzo

**Affiliations:** 1 Department MeSVA, University of L’Aquila, L’Aquila, Italy; 2 Unità Operativa Semplice Dipartimentale di Pronto Soccorso Odontoiatrico, con annessa Unità di Odontoiatria Conservativa, University of Tor Vergata, Roma, Italy; 3 University Vita-Salute San Raffaele, Via Olgettina 60, 20132 Milano, Italy

**Keywords:** Palatal expansion, Periodontal indices, Plaque index, Papillary bleeding index, Probing pocket depth

## Abstract

**Objectives:**

The aim of this pilot study was to evaluate the periodontal effects during rapid palatal expansion (RPE) or slow palatal expansion (SPE) and to compare them by means of some clinical indices, in order to establish the possible differences and advantages of one of these treatments in periodontal terms.

**Methods:**

10 patients (aged 6 to 7 years; average age 6.3 years) were submitted to RPE treatment and other 10 patients (aged 6 to 8 years, average age 6.3 years) to SPE treatment. They were treated with the Haas expander. The selected clinical indices (plaque index, PI; papillary bleeding index, PBI; probing pocket depth, PPD) were collected three times during the treatment (t_0_, detected 7 days after the periodontal prophylaxis, at the beginning of the active orthodontic therapy; t_1_, detected during the active therapy; t_2_, detected after retention). All measurements were performed by the same examiner. The protocol was approved by the ethics committee.

**Results:**

The effects of the prophylaxis were excellent to control inflammation and dental plaque before the beginning of the orthodontic-orthopaedic treatment, as in both the two groups, the PI and the PBI values were equal to 0.

In the group receiving slow expansion, the PPD remained unchanged from t_0_ to t_1_, while it significantly increased from t_0_ to t_1_ in the group of rapid expansion. At t_2_ the values of the two groups returned to be overlapping.

**Conclusions:**

Both rapid and slow expansion treatments present potential irritation effect (increase of PI index and PBI index) on the periodontium, suggested by the significant increase of PI and PBI from t_0_ to t_1_ in both the two groups; therefore prophylaxis and periodic controls are very important. There are no long-term benefits that might be referred unequivocally to one of the two treatments in terms of periodontal consequences, as demonstrated by the lack of significant differences between the two groups at t_2_.

## Introduction

Over the past years there has been an appreciable increase of the orthodontic treatments, but the clinicians must not forget that orthodontic therapies can cause side-effects affecting the periodontal tissues.

The oral cavity is a rich ecosystem with a plethora of microorganisms. Plaque bacteria are the major factor in the onset and progression of periodontal disease and caries, but these are really multifactorial diseases, and there are situations which comprise what has been termed ‘ecological stress’, causing the shift of the microbiological balance, so creating conditions conducive to the growth, and appearance of cariogenic and/or periodontopathic bacteria [1]. The different components of a fixed orthodontic system may contribute to the shift in the balance of the oral ecology. Correlations have been observed between orthodontic treatment and its effects on periodontal tissues [2-4]. A periodontal interaction subsequent to rapid palatal expansion [5] has also been described in terms of fluctuation of the inflammatory mediators, such us interleukin-1β (IL-1β) e β-glucoronidase (βG) levels in the gingival crevicular fluid (GCF) of the first maxillary molars during rapid palatal expansion [6].

The effect of an orthodontic force also depends from the type of appliance [7].

Except for the type of the tested expansion therapy, none of the other examined orthodontic variables showed a statistically significant influence on the periodontal tissues [7].

Although the phenomenon of orthodontic movement is similar to inflammation, but relatively aseptic [8], additional inflammation, such as the one induced by plaque accumulation, should be avoided during orthodontic and orthopaedic treatment [6].

As the orthodontic appliances certainly facilitate the establishment of significant bacterial colonies that can alter the environment of oral cavity [9], the clinician must ensure that there are no active inflammatory processes in the periodontium before starting all orthodontic treatments. It is recommended to educate and motivate the patient to oral hygiene; to perform eventual periodontal prophylaxis; to carefully choose the typology of appliance; and to carry out periodic medical controls at shorts intervals [10].

The periodontal prophylaxis program depends on: patients age; clinical oral and systemic conditions; patients collaboration; and family and social environment.

This pilot study was aimed to evaluate the periodontal health clinical indices in patients subjected to rapid and slow palatal expansion (using the Haas expander), and compare them establishing the possible differences and advantages of one of these treatments.

## Materials and methods

Due to the small cohort of this study, this can be considered only a pilot study.

It was performed at the Department of Oral Health, University of L’Aquila, Italy.

10 patients (5 males and 5 females) (Group I), aged 6 to 7 years (average age 6.3 years), were subjected to rapid palatal expansion (RPE) using the Haas expander. A second group included 10 patients (4 males and 6 females) (Group II), aged 6 to 8 years, average age 6.3 years, that were subjected to slow palatal expansion (SPE) with the same appliance activated in different times and ways.

The subjects were enrolled in the sample from april 2011 to november 2011. The parents of the participants agreed (informed) to partecipate to this study.

The following inclusion criteria were observed: no dental diseases (caries, fractures, granulomas, etc.), a constriction of the upper jaw, that needed the palatal expansion appliance mounted on bands in order to be treated; good general health; probing depth values not exceeding 3 mm in the whole dentition; no radiographic evidence of periodontal bone loss. In addition, patients showed normal gingival biotype [11,12].

The gingival biotype was objectively assessed using a metal periodontal probe in the sulcus to evaluate gingival tissue thickness: a thin biotype was recorded when the tip of the probe was visible through the gingiva; a normal biotype was assessed when the tip of the probe was not visible. See text for details [11,12].

Periodontal condition of the patients prior to starting the study was analyzed in detail and reported in Table 1.

**Table 1:** Periodontal conditions of the patients prior to starting the study

**Group I**	
(5 males and 5 females; average 6.3; range from 6 to 7 years old)	
Patient 1.	Probing depth values not exceeding 3 mm in the whole dentition; no radiographic evidence of periodontal bone loss; normal gingival biotype.
Patient 2.	Probing depth values not exceeding 3 mm in the whole dentition; no radiographic evidence of periodontal bone loss; normal gingival biotype.
Patient 3.	Probing depth values exceeding 3 mm only in two sites; no radiographic evidence of periodontal bone loss; normal/thin gingival biotype.
Patient 4.	Probing depth values exceeding 3 mm only one site in the whole dentition; no radiographic evidence of periodontal bone loss; normal gingival biotype.
Patient 5	Probing depth values not exceeding 3 mm in the whole dentition; no radiographic evidence of periodontal bone loss; normal gingival biotype.
Patient 6.	Probing depth values not exceeding 3 mm in the whole dentition; no radiographic evidence of periodontal bone loss; normal gingival biotype.
Patient 7.	Probing depth values exceeding 3 mm only in two sites; no radiographic evidence of periodontal bone loss; normal/thin gingival biotype.
Patient 8.	Probing depth values not exceeding 3 mm in the whole dentition; no radiographic evidence of periodontal bone loss; normal gingival biotype.
Patient 9.	Probing depth values not exceeding 3 mm in the whole dentition; no radiographic evidence of periodontal bone loss; normal gingival biotype.
Patient 10.	Probing depth values not exceeding 3 mm in the whole dentition; no radiographic evidence of periodontal bone loss; normal gingival biotype.
**Group II**	
(4 males and 6 females; average 6.3; range from 6 to 8 years old)	
Patient 1.	Probing depth values exceeding 3 mm only in one site; no radiographic evidence of periodontal bone loss; normal gingival biotype.
Patient 2.	Probing depth values exceeding 3 mm only in one siteno radiographic evidence of periodontal bone loss; normal gingival biotype.
Patient 3.	Probing depth values not exceeding 3 mm in the whole dentition; no radiographic evidence of periodontal bone loss; normal gingival biotype.
Patient 4.	Probing depth values not exceeding 3 mm in the whole dentition; no radiographic evidence of periodontal bone loss; normal gingival biotype.
Patient 5.	Probing depth values exceeding 3 mm only in two sites; no radiographic evidence of periodontal bone loss; normal/thin gingival biotype.
Patient 6.	Probing depth values not exceeding 3 mm in the whole dentition; no radiographic evidence of periodontal bone loss; normal/thin gingival biotype.
Patient 7.	Probing depth values not exceeding 3 mm in the whole dentition; no radiographic evidence of periodontal bone loss; normal gingival biotype.
Patient 8.	Probing depth values not exceeding 3 mm in the whole dentition; no radiographic evidence of periodontal bone loss; normal gingival biotype.
Patient 9.	Probing depth values exceeding 3 mm only in two sites; no radiographic evidence of periodontal bone loss; normal gingival biotype.
Patient 10.	Probing depth values not exceeding 3 mm in the whole dentition; no radiographic evidence of periodontal bone loss; normal gingival biotype.

Before the start of the expansion the patients received periodontal prophylaxis treatment, which included scaling, education and motivation to oral hygiene.

The patients were treated with the same appliance but using different clinical procedure. The Haas expander was anchored to the second deciduous molars with bands and bonded to the deciduous canines using acrylic resin [13,14].

After a week, in the group of patients receiving RPE (Group I), the jackscrew of the expander was activated once (0.25 mm) from the operator and once by the patient or his/her parent (total daily activation, 0.5 mm). Then the jackscrew was activated once in the morning and once in the evening (0.5 mm every 24 hours) for 20 days. After the active therapy, the appliance was stabilized by blocking the screw and there were 5 months of retention therapy [15].

At the same time, patients in Group II were subjected to slow palatal expansion. The jackscrew of the Haas expander was activated twice a week (total activation, 0.5 mm per week). After 3 months of active therapy, there was set a 3 months retention period with the same appliance [15].

The following periodontal clinical indices were used to assess the gingival and periodontal health:

– plaque index (PI, Silness e Löe) [16]: the measurement of the state of oral hygiene by Silness-Löe plaque index is based on recording both soft debris and mineralized deposits on the teeth. Each of the four surfaces of the teeth (buccal, lingual, mesial and distal) is given a score from 0–3. The scores from the four areas of the tooth are added and divided by four in order to give the plaque index for the tooth.

– papillary bleeding index (PBI, Saxer e Muhlemann) [17]: this index permits both immediate evaluation of the patient’s gingival condition and his motivation, based upon the actual bleeding tendency of the gingival papillae. A periodontal probe is inserted into the gingival sulcus at the base of the papilla on the mesial aspect, and then moved coronally to the papilla tip. This is repeated on the distal aspect of the papilla. The intensity of any bleeding is recorded (0–4 scores).

– probing pocket depth (PPD).

The probing pocket depth was measured in 3 vestibular sites (mesio-buccal, buccal and disto-buccal) and 3 palatal sites (mesio-palatal, palatal and disto-palatal), at the level of the first upper molars, left and right. An standard WHO clinical periodontal probe was used. To standardize the procedure, the probe was inserted until its tip encountered the resistance of the junctional epithelium that forms the base of the sulcus. The pressure exerted with the probe tip against the junctional epithelium was between 10 and 20 grams. A sensitive scale that measures weight in grams was used to standardize the probing pressure. One author (SM) completed the experimental procedures.

The repeatability of the procedure was evaluated with the Intraclass Correlation Coefficient (ICC) applied to double measurements recorded from 5 subjects two times, at a distance of 30 minutes between the first and the second evaluations.

The ICC is the proportion of the total variance within the data that is explained by the variance between the two evaluations. The value of the ICC ranges from 0 to 1, where as the ICC approaches a value of one then we see a perfect agreement between the evaluations, and as the ICC approaches a value of zero then we see no agreement between the evaluations.

In the Group I (RPE), all these indices were detected in three stages:

– T_0_, detected 7 days after the periodontal prophylaxis (at the beginning of the active orthodontic therapy);

– T_1_, detected after 20 days of active therapy;

– T_2_, detected after 5 months of retention therapy.

In the Group II, the collected data corresponded to:

– T_0_, detected 7 days after the periodontal prophylaxis;

– T_1_, detected during clinical control after 3 months of active therapy;

– T_2_, detected after 3 months of restraint.

All measurements were performed by the same examiner.

### Data analysis

A descriptive statistical analysis was conducted. The ANOVA evaluation and Tukey’s post-hoc analyses were conducted to evaluate the intra-group differences among t_0_, t_1_ and t_2_. The unpaired samples Student’s T test was used to evaluate between groups differences at t_0_, t_1_ and t_2_. The significance level was set at 95%.

## Results

Demographic data and periodontal conditions prior to starting the study are reported in Table 1.

The Interclass Correlation Coefficient (ICC) is reported in Table 2.

**Table 2:** Intra-observer method error calculated with Intraclass Correlation Coefficient (5 subjects)

	**T0**	**T1**	**Intraclass Correlation Coefficient**
**PI**	0	0.3	ICC: 0.9
**PBI**	0	0.2	ICC: 0.8
**PPD (mm) 1.6**	2.5	2.6	ICC: 0.9
**PPD (mm) 2.6**	2.5	2.5	ICC: 0.9

As exposed in Table 3 at t_0_, the effects of the periodontal prophylaxis were excellent in controlling periodontal inflammation and dental plaque before the beginning of the orthodontic and orthopaedic treatment, as in both the two groups, the PI and the PBI values were equal to 0.

**Table 3:** Main results of periodontal indices

	**Group I**				**Group II**				**Difference between the 2 groups at T1**	**Difference between the 2 groups at T2**
	**T0**	**T1**	**Differences between T0 and T1**	**T2**	**T0**	**T1**	**Differences between T0 and T1**	**T2**		
**PI**	0	0.9	t:9; p < 0.001	0.5	0	0.7	t:7; p < 0.001	0.5	NS	NS
**PBI**	0	0.9	t:9; p < 0.001	0.3	0	0.7	t:7; p < 0.001	0.3	NS	NS
**PPD 1.6**	2.5	2.9	t:2.6; p < 0.05	2.9	2.4	2.4	NS	2.9	NS	NS
**PPD 2.6**	2.5	2.9	t:2.6; p < 0.05	3	2.5	2.4	NS	2.8	NS	NS

PI: plaque index (Silness e Loe) [16][13].

PBI: papillary bleeding index (Saxer e Muhlemann) [17][14].

PPD: probing pocket depth (mm).

T0: 7 days after the periodontal prophylaxis (at the beginning of the active orthodontic therapy).

T1: after 20 days of active therapy.

T2: after 5 months of retention therapy.

Regarding the PPD of the first maxillary molars, for simplicity, the data detected in the palatal areas were assumed as reference values.

### PI index

In the Group I, the average PI was 0 at t_0_, 0.9 at t_1_ and 0.5 at t_2_.

In the Group II, the average PI was 0 at t_0_, 0.7 at t_1_ and 0.5 at t_2_.

At t_2_ the two groups overlapped the same value.

### PBI index

In the Group I, the average PBI passed from 0 at t_0_, to 0.9 at t_1_, and to 0.3 at t_2_.

In the Group II, the PBI passed from 0 at t_0_ to 0.7 at t_1_, and to 0.3 at t_2_.

At t_2_ the two groups overlapped the same value.

### PPD index

In the Group I the average PPD in the right maxillary molar passed from 2.5 at t_0_ to 2.9 (at t_1_ and t_2_); in the left maxillary molar it passed from 2.5 at t_0_ to 2.9 at t_1_, and 3 at t_2_ (Table 3).

In the Group II, the PPD in the right maxillary molar was 2.4 at t_0_ and t_1_ and passed to 2.9 at t_2_; the PPD of the left maxillary molar passed from 2.5 at t_0_ to 2.4 at t_1_ and 2.8 at t_2_ (Table 3).

## Discussion

In this study, the clinical indices of periodontal health were selected as they are easily assessable by an orthodontist for a regular periodic monitoring of periodontal health during the orthodontic treatment.

As exposed in Table 3, after 7 days of periodontal prophylaxis and before the beginning of the orthodontic-orthopaedic therapy (t_0_), the values of the periodontal indices indicate an excellent state of periodontal health for the patients in both the groups. The PI and PBI were 0 in both the groups. The PPD values nearly overlap.

In both the groups there was an evident increase of the values of periodontal indices (PI and PBI) from t_0_ e t_1_.

These increases of PI and PBI indices were statistically significant with p <0.001 in both the two Groups, suggesting that they were not due to chance, but to a real difference in periodontal conditions during the orthodontic treatment.

These observations suggest potential irritation effects of the palatal expander on clinical indices of periodontal health, as also demonstrated for fixed orthodontic brackets [18].

In particular, in Group I the increase of values from t_0_ to t_1_ regarding PI and PBI (0.9) was more pronounced than in Group II.

In addition, in the Group I, the differences between the averages of the PPD from t_0_ to t_1_ were both statistically significant for the left and the right molars (PPD: 2.9; p <0.05) (Table 3).

On the contrary, in Group II, at t_1_ the PPD did not increase in both the right and the left molars, and there was also a slight decrease of the relative average PPD value of the left molar, from 2.5 to 2.4.

Although these differences, the periodontal conditions remained in the periodontal health “range” at t_1_ in both the Groups.

These observations seemed to suggest a potentially more dangerous effects of the rapid expansion (Group I) respect to slow expansion (Group II).

This is in accordance with studies about biological responses of these appliances, which suggested that the application of slow palatal expansion in areas of periosteal growth allows normal arch dimensions to develop at any age without undue tipping of the abutment teeth [19,20] potentially avoiding periodontal dangerous effects on the abutment teeth.

This has also been demonstrated for a tissue-borne, fixed, acrylic plate appliance used for RPE, respect to a quad-helix appliance used for SPE in another study [7], although the results of these two study are not directly comparable, because of the different employed appliances (acrylic or metal fixed plate used for RPE) and different recorded variables (clinical and radiographic variables).

In this study, the better state of periodontal health at t_1_ observed in the Group II - emphasized by PPD of the left and right molars both equal to 2.4 – could be due to a greater number of controls during SPE, associated to the longer period of active therapy with SPE.

Results seem to mean that the SPE procedure provides for better monitoring of the clinical indices of periodontal health than the procedure of RPE. Therefore, during the RPE the clinician should pay more attention to the control of periodontal health through regular periodic monitoring, as also suggested in literature for other dental treatments [21].

At t_2_ the data of periodontal indices (Table 3) of the two Groups nearly overlapped again, with slight differences, and were in the periodontal health “range” because in both the Groups the PI was 0.5 and the PBI was 0.3.

At t_2_ the PPD of the right molar was the same in both groups and equal to 2.9. The PPD of the left molar in Group I was slightly higher (value 3) than in Group II (value 2.8).

Thus in conclusion, there were not important differences in the periodontal health after the retention between RPE and SPE.

The results indicate that both RPE and SPE exhibit minimal differences in periodontal condition after retention (t_2_), and the state of the periodontium is good in all groups, although a potential more dangerous effect of RPE during the active treatment. Although the average differences were clinically small, individual variations were evident among few patients subjected to a more pronounced periodontal breakdown in the central side of the first molars. Most of them in the group receiving RPE. Due to the small cohort of this study, it can be considered only a pilot study; thus certain clinical conclusions are not possible. In addition, as the attachment loss was not evaluated, it is not clear – on the base of these results - whether the appliance being in place for almost up to 3 months, would bring about gingival inflammation, potentially giving rise to a pseudo-pocket formation; this factor could have potentially affected the outcome of the study.

Finally, except for the type of the tested expansion therapy, none of the other examined orthodontic variables seemed to have an influence on the periodontal tissues.

## Conclusions

According to the data discussed above, in view of the limits of the pilot study design, we can conclude that:

– periodontal prophylaxis appear successful in the control of periodontal health;

– the palatal expander seems to influence periodontal health: both rapid and slow expansion treatments present potential irritation effect (increase of plaque and bleeding) on the periodontium, suggested by the significant increase of PI and PBI from t_0_ to t_1_ in both the two groups;

– the difference between the treatment of RPE and SPE during the active therapy (t_1_), can be attributed with high probability to the greater number of clinical controls performed during the SPE treatment;

– there are no substantial differences in the long term (t_2_) in the periodontal health, after the period of retention, between the treatment of RPE and SPE;

– there are no important advantages that can be unequivocally point to one of the two treatments in periodontal terms.

– PI, PBI and PPD can be used for measurement of periodontal status during the palatal expansion procedure;

The clinically relevant conclusion is that the palatal expansion procedure can affect periodontal health; therefore the clinician should pay more attention to the control of periodontal health through regular periodic monitoring.

Further related studies with a greater sample are recommended to better clarify these relationships.

### Ethical approval

This protocol was approved by the Ethic Committee of the University of L’Aquila, in accordance with the principles of the D.Lgs. n. 200 del 6.11.2007, nel D.Lgs. n. 211 del 2003 e nel D.M. 12.5.2006 (Italian Law) with which the Legislator has regulated the establishment of ethics committees for clinical trials of medicinal products, and excluded from the scope of this legislation the so-called non-interventional drug trials (or observational studies), and the experimentation on the People that do not involve the use of drugs or involving human biological materials (tissues, cells, etc.). For protocols like this - non-interventional drug trials (or observational studies) - the Ethics Committee of the University of L’Aquila has recently instituted an apposite section called “Ethics Committee of the University for the evaluation of epidemiological and non-pharmacological observational studies” who gave positive think about the protocol (DR 206/2013, sit 16-7-2013).

## Competing interests

The authors declare that they have no competing interests.

## Authors’ contribution

SM organized the protocol of research; EM coordinated the recording of data; FA helped in the recording of data; VC wrote and organized the manuscript; FP helped in the recording of data; SDM helped in the recording of data; ST organized the data, made the statistical analysis, the analysis of results and wrote the manuscript; and GM wrote and organized the manuscript and the interpretation of results. All the authors read and approved the final manuscript.

## References

[B1] Marsh PD Are dental diseases examples of ecological catastrophes? Microbiology 2003 149 279 294 10.1099/mic.0.26082-0 12624191

[B2] Bishara SE Staley RN Maxillary expansion: clinical implications Am J Orthod Dentofacial Orthop 1987 91 3 14 10.1016/0889-5406(87)90202-2 3541577

[B3] Grieve WG 3rd Johnson GK Moore RN Reinhardt RA DuBois LM Prostaglandin E (PGE) and interleukin-1 beta (IL-1 beta) levels in the gingival crevicular fluid during human orthodontic tooth movement Am J Orthod Dentofacial Orthop 1994 105 369 374 10.1016/S0889-5406(94)70131-8 8154462

[B4] Ousehal L Lazrak L Es-Said R Hamdoune H Elquars F Khadija A Evaluation of dental plaque control in patients wearing fixed orthodontic appliances: a clinical study Int Orthod 2011 9 1 140 155 21277846 10.1016/j.ortho.2010.12.013

[B5] Rosa M Cozzani M Espansione rapida del mascellare superiore nel rispetto del parodonto 1995 Milano, Italy: Schede di aggiornamento S.I.D.O

[B6] Tzannetou S Efstratiadis S Nicolay O Gribic J Lamster I Comparison of levels of inflammatory mediators IL-1 and G in gingival crevicular fluid from molars, premolars, and incisors during rapid palatal expansion Am J Orthod Dentofacial Orthop 2008 133 5 699 707 10.1016/j.ajodo.2006.03.044 18456143

[B7] Greenbaum KR Zachrisson BU The effect of palatal expansion therapy on the periodontal supporting tissues Am J Orthod 1982 81 1 12 21 10.1016/0002-9416(82)90283-4 6758588

[B8] Garlet TP Coelho U Repeke CE Silva JS Cunha Fde Q Garlet GP Differential expression of osteoblast and osteoclast chemmoatractants in compression and tension sides during orthodontic movement Cytokine 2008 42 3 330 335 10.1016/j.cyto.2008.03.003 18406624

[B9] Ro R Oliveira CA dos Santos-Pinto A Jordan SF Zambon JJ Cirelli JA Haraszthy VI Clinical and microbiological studies of children and adolescents receiving orthodontic treatment Am J Dent 2010 23 6 317 323 21344829

[B10] Zachrisson BU Clinical Implications of recent orthodontic-periodontic research findings Semin Orthod 1996 2 1 4 12 10.1016/S1073-8746(96)80034-X 9161278

[B11] Kan JY Rungcharassaeng K Umezu K Kois JC Dimen- sions of peri-implant mucosa: an evaluation of maxillary anterior single implants in humans J Periodontol 2003 74 4 557 562 10.1902/jop.2003.74.4.557 12747463

[B12] Esfahrood ZR Kadkhodazadeh M Talebi Ardakani MR Gingival biotype: a review 2013 Gen Dent 14 17 http://www.agd.org 23823337

[B13] Rosa M Expansão rapida da maxilla na dentizão mista sem incluir os dentes permanentes: indicazoes momento oportuno Rev Clin Ortod Dental Press 2011 10 5 106 118

[B14] Cozzani M Rosa M Cozzani P Siciliani G Deciduous dentition-anchored rapid maxillary expansion in cross bite and non-cross bite mixed dentition patients: reaction of the permanent first molar Prog Orthod 2003 4 2 15 22 12887575 10.1034/j.1600-9975.2002.02034.x

[B15] Huynh T Kennedy DB Joondeph DR Bollen AM Treatment response and stability of slow maxillary expansion using Haas, hyrax, and quad-helix appliances: a retrospective study Am J Orthod Dentofacial Orthop 2009 136 331 339 10.1016/j.ajodo.2007.08.026 19732666

[B16] Silness J Loe H Periodontal disease in pregnancy. II. correlation between oral hygiene and periodontal condition Acta Odontol Scand 1964 22 121 135 10.3109/00016356408993968 14158464

[B17] Saxer UP Muhlemann HR Motivation and Education SSO Schweiz Monatsschr Zahnheilkd 1975 85 9 905 919 1059253

[B18] Mummolo S Marchetti E Giuca MR Gallusi G Tecco S Gatto R Marzo G In-office bacteria test for a microbial monitoring during the conventional and self-ligating orthodontic treatment Head Face Med 2013 9 7 10.1186/1746-160X-9-7 23375053 PMC3563582

[B19] McAndrew JR The continuous force control system 1985 Carlsbad, CA: Lancer Technical Report, Lancer Pacific

[B20] Hicks EP Slow maxillary expansion: A clinical study of the skeletal vs. dental response to low magnitude force Am J Orthod 1978 73 121 141 10.1016/0002-9416(78)90183-5 343597

[B21] Marchetti E Monaco A Procaccini L Mummolo S Gatto R Tetè S Baldini A Tecco S Marzo G Periodontal disease: the influence of metabolic syndrome Nutr Metab (Lond) 2012 9 1 88 10.1186/1743-7075-9-88 23009606 PMC3499456

